# Association of heel bone mineral density with incident dementia among ageing adults: a population-based study from the UK Biobank

**DOI:** 10.1007/s40520-025-03100-w

**Published:** 2025-07-16

**Authors:** Jun Lu, Frank Mastaglia, Andrew Chi Pang Tai, Max K. Bulsara, William G. Blakeney, Charles A. Inderjeeth, Minghao Zheng, Jun Yuan

**Affiliations:** 1 Perron Institute for Neurological and Translational Science, University of Western Australia, 8 Verdun St, Nedlands, WA 6009 Australia; 2 Centre for Translational Orthopaedic Research, School of Biomedical Sciences, University of Western Australia, 35 Stirling Highway, Perth, WA 6009 Australia; 3 Institute for Health Research, University of Notre Dame Australia, 23 High St, Fremantle, WA 6160 Australia; 4 Department of Orthopaedic Surgery, Royal Perth Hospital, Wellington Street, Perth, WA 6000 Australia; 5 Department of Surgery, Medical School, University of Western Australia, 35 Stirling Highway, Perth, WA 6009 Australia; 6 Sir Charles Gairdner Osborne Park Health Care Group, Hospital Ave, Gerontorheumatology, Nedlands, WA 6009 Australia; 7 Rheumatology Group, Medical School, University of Western Australia, 35 Stirling Highway, Perth, WA 6009 Australia

**Keywords:** Dementia, Estimated bone mineral density, Osteoporosis, Cohort studies, UK Biobank

## Abstract

**Background:**

Osteoporosis and dementia are two common disorders mainly affecting ageing population, and heel estimated bone mineral density (eBMD) measured by quantitative ultrasound (QUS) has been demonstrated to be a reliable and non-invasive method for assessing skeletal health. The aim of this study is to determine the association between eBMD and incident dementia in older adults.

**Methods:**

This retrospective cohort study employs UK Biobank data of 131,030 adults aged ≥ 60 years without dementia at baseline. Cox proportional-hazards models were used to investigate the association between eBMD and incident dementia, with the C-index evaluating the discriminative potential of eBMD.

**Results:**

Among participants (52.1% women, median [IQR] age was 64 [62–66] years), there were 4,572 cases (3.5%) of incident dementia. Minimal model showed that participants with low eBMD (< 0.467 g/cm^2^) had a 14% increase in the rate of dementia incidence (HR 1.14, 95% CI 1.06–1.23; *P* <.001), and each standard deviation (SD) decrease in eBMD was associated with a 49% increase in dementia risk (HR 1.49, 95% CI 1.19–1.86; *P* <.001). Such association remained significant after further adjustment for potential confounders. Stratified analyses revealed that lower eBMD increased dementia risk in male participants (HR 1.17, 95% CI 1.06–1.31; *P* =.003) and in participants with neutral (HR 1.18, 95% CI 1.05–1.33; *P* =.007) or low genetic risk (HR 1.36, 95% CI 1.01–1.83; *P* =.04). Sensitivity analyses showed similar results. However, discriminative analyses showed minimal improvement after adding eBMD to dementia prediction models.

**Conclusion:**

Lower heel eBMD is independently associated with increased dementia risk among older adults.

**Supplementary Information:**

The online version contains supplementary material available at 10.1007/s40520-025-03100-w.

## Introduction

Dementia and osteoporosis are two common age-related diseases predominantly affecting older adults, with an estimated 55 million and 500 million cases respectively reported around the globe [1, 2]. The two conditions frequently coexist and clinical outcomes have reported that people with osteoporosis are more likely to experience cognitive decline [3, 4], which is the precursor of dementia. Notably, our previous cohort study along with a recent in vivo investigation, has highlighted the involvement of sclerostin, a bone-derived protein, in the pathogenesis of Alzheimer’s disease (AD), a major form of dementia [5, 6]. Given this, it is plausible that bone health outcomes are associated with the onset and progression of dementia during ageing. As there is currently no cure for dementia, identifying individuals at substantial risk of developing dementia could facilitate earlier diagnosis of dementia and enable patients and their families to implement lifestyle modifications to slow disease deterioration. Decreasing bone mineral density (BMD) is one of the key pathological features of osteoporosis, and accumulating evidence has demonstrated the association between low BMD and increased risk of dementia in later life [7, 8, 9, 10, 11, 12]. This makes BMD a promising indicator for screening individuals at risk of incident dementia. However, existing studies only investigated BMD measured by dual-energy X-ray absorptiometry (DXA) at skeletal sites such as the femoral neck and lumbar spine, and the relationship between DXA-BMD and risk of dementia differs depending on the specific skeletal site measured [3, 10, 13]. Additionally, the majority of studies investigated only relatively small sample sizes (i.e., < 5,000 participants [9, 10]) or were cross-sectional in nature [8], resulting in limited reliability of the findings.

The alternative technique of quantitative ultrasound (QUS), which measures estimated BMD (eBMD) in the heel (calcaneus) has been demonstrated to be a reliable and non-invasive method for assessing skeletal health [14]. While several studies have shown low to modest correlations between QUS variables and DXA-BMD regardless of sex and region [15, 16], calcaneal QUS can produce reliable absolute BMD measurements, making it a practical option for osteoporosis screening and predicting fractures [17, 18, 19, 20]. Additionally, the heel eBMD has the advantage of simplicity of measurement, no radiation exposure, and low cost compared to DXA-based techniques. However, the association between eBMD and incident dementia among older adults is as yet undetermined. Given the link between BMD and cognitive impairment, it is hypothesized that eBMD correlates with incident dementia and can serve as a screening tool for late-life dementia.

The purpose of this study was to use data from a population-based cohort of more than 130,000 participants over 15 years of follow-up, to investigate the association of eBMD with incident dementia among an ageing population. We first determined whether lower eBMD is associated with an increased risk of developing all-cause dementia in later life. Secondly, we examined whether sex and *APOE* status, which are two major risk factors for dementia, modify the relationship between eBMD and incident dementia. Finally, we determined if eBMD provided added value for incident dementia discrimination. This study provides further evidence supporting the interaction between bone and brain health, and adds to the literature that aims to identify individuals at risk of dementia most likely to benefit from preventative strategies targeting bone.

## Methods

### Study population and design

The UK Biobank is a population-based cohort of more than half-a-million participants recruited in 1 of 22 assessment centres across the United Kingdom between 2006 and 2010 [21]. To ensure the study population consisted of individuals at risk of developing dementia over the follow-up period, the present analysis was restricted to individuals aged 60 years or older at baseline. Participants with prevalent self-reported or diagnosed cognitive impairment or dementia at baseline were excluded. We also excluded participants without valid measurement of eBMD at baseline or with invalid data for any covariates. Participants were followed up from the date of enrolment until their first dementia diagnosis (incident dementia), death, lost to follow-up date, or to the latest date of data update by UK Biobank (30/09/2024), whichever came first.

### Measurement of QUS and eBMD

Heel eBMD was derived from QUS measurement of the calcaneus. Baseline QUS data were generated by measuring the left calcaneus from 2007 to mid-2009. Measurement of the right calcaneus was recorded in cases where the left was missing or deemed unsuitable. The calcaneal QUS was performed using the Sahara Clinical Bone Sonometer [Hologic Corporation (Bedford, MA, USA)] and the data was automatically inputted with Vox software. QUS outcomes were manually inputted by the attending healthcare technician or nurse when direct input failed. Speed of sound (SOS) and broadband ultrasound attenuation (BUA) were recorded at assessment, and eBMD was derived as a linear combination of SOS and BUA using the equation: [eBMD = 0.002592 * (BUA + SOS) − 3.687]. More detailed information on the methods is available on the UK Biobank website (Resource 100248, Ultrasound Bone Densitometry, Version 1.0).

### Ascertainment of incident dementia

All-cause dementia was ascertained based on inpatient hospital records and death registry data. Primary and secondary hospital diagnoses (hospital records) and causes of death (death register) for participants with dementia were identified using the International Classification of Diseases, Ninth Revision (ICD-9) and ICD-10 coding systems. The codes used to ascertain dementia were selected and validated by the UK Biobank outcome adjudication group (Resource 8319, version 2.0, Jan 2022) (Supplementary File, Table S1).

### Covariates

Potential covariables associated with bone health, dementia or both were selected according to literature evidence, including sociodemographic, lifestyle and genetic factors [22, 23]. Baseline covariables of sociodemographic and lifestyle included age (in years), sex (female and male), body mass index (BMI, kg/m^2^), education level (higher education, secondary education, vocational education and other), ethnicity (classified based on self-report and dichotomized as White/non-White), socioeconomic status (assessed with the Townsend deprivation index [24]), smoking status (never, previous and current), alcohol intake status (never, previous and current), physical activity level (< 150 min/week as low, ≥ 150 min/week as high) and chronic conditions (physician diagnosis of stroke, diabetes, hypertension, and two bone-specific disorders including arthrosis and rheumatoid arthritis).

*APOE* allele status was considered to be a potential genetic confounder. The Affymetrix UK BiLEVE Axiom Array and the Affymetrix UK Biobank Axiom Array were employed for genotyping by UK Biobank, and *APOE* allele status was determined based on the variants at the rs429358 and rs7412 single nucleotide polymorphisms (SNPs) [25]. Three main *APOE* alleles (*ε*4, *ε*3, and *ε*2) are defined, leading to six common genotypes (*ε*4*ε*4, *ε*4*ε*3, *ε*4*ε*2, *ε*3*ε*3, *ε*2*ε*3 and *ε*2*ε*2). Previous evidence has suggested that the *ε*3 allele of the *APOE* gene is “risk neutral”, the *ε*2 allele has neuroprotective effects, and the *ε*4 allele contributes to a higher risk of developing dementia [26, 27, 28]. In addition, *ε*2*ε*4 is found to be associated with cognitive decline in older adults [29]. Ambiguous genotype *ε*2*ε*4/*ε*1*ε*3 was coded as *ε*2*ε*4 since the *ε*1 allele is extremely rare and the role of *ε*1 allele in cognitive function is unclear [30, 31]. Therefore, we categorized the participants into low (*ε*2*ε*2, *ε*2*ε*3), neutral (*ε*3*ε*3), and high (*ε*4*ε*4, *ε*4*ε*3, *ε*4*ε*2) dementia risk groups. There were occasions where the genotype could not be unambiguously determined as the SNP data were unphased, and these participants were categorized into an unsure group. Details of the *APOE* SNP alleles and genotype coding are provided in Supplementary File (Table S2).

### Statistical analysis

Baseline characteristics of the participants were summarized across dementia status as percentage for categorical variables, mean ± SD for normally distributed continuous variables, and median (interquartile range, IQR) for non-normally distributed continuous variables.

To assess the association between eBMD and incident all-cause dementia, we first assessed the potential nonlinearity by using likelihood ratio test. The Cox proportional-hazard models with only a linear term were compared against models with cubic spline terms with three knots (10th, 50th and 90th percentiles) using RMS package in R. The Akaike information criterion was used to determine whether linear or nonlinear terms provided the best model fit. Shapes of nonlinear associations were estimated using restricted cubic splines with the reference value set at the median of eBMD, ensuring the results were less sensitive to potential outliers or extreme values in the exposure distribution.

Subsequently, tertile categories of eBMD were used as an exposure, and effects of eBMD on incident dementia were also assessed by treating eBMD as a continuous variable expressed as per 1SD decrease. Person-years were calculated by multiplying the number of people by the time each person was followed up. Cox models minimally adjusted for age, sex and *APOE* status, with attained age as the timescale were firstly used to estimate the hazard ratios (HRs) and 95% confidence intervals (CIs) in relation to the low and high levels of exposure, using the medium level of eBMD as the reference. We next evaluated the fully adjusted models adjusted for age, sex and additional covariates including body mass index, education level, ethnicity, socioeconomic status, smoking status, alcohol intake status, physical activity level, chronic conditions. The Kaplan-Meier cumulative incidence curves were used to map group differences in eBMD tertiles for dementia. Since death is a strong competing risk for the incidence of dementia, competing risk regression model was implemented for computing the cumulative incidence for each group using “cmprsk” package in R. The proportional-hazards assumption in all models was checked by Schoenfeld residuals and satisfied.

The interaction between eBMD with sex and *APOE* status in the fully adjusted model was examined using Wald test to determine if potential effect modification exists, and stratification analysis by sex and *APOE* status was performed. In the sensitivity analyses, follow-up time was stratified (≤ 5 years, 5–10 years, and ≥ 10 years) to determine the change of the aforementioned risk of incident dementia. Furthermore, we conducted stratified analyses to determine the effect modification by age groups (60–65 years and over 65 years). Serum phosphate homeostasis is mainly regulated by fibroblast growth factor 23 (FGF-23), which is primarily produced by osteocytes [32]. To explore the robustness of the association between eBMD and incident dementia, we conducted additional adjustment for serum phosphate levels, given prior evidence linking phosphate metabolism to cognitive decline [33]. Additional analysis was performed using the dataset with missing covariate data imputed to determine whether the main analyses were biased. Multiple imputations by chained equations with 40 imputations (based on the proportion of observations with missing values [34]) were used to impute missing variables using the MICE package in R. The proportion of missing data for each variable are listed in Supplementary File (Table S3).

To evaluate the value of eBMD on incident dementia discrimination over the max available follow-up time for the participants, C-index from Cox models were calculated using the Hmisc R package with the data before imputations. Comparisons of differences between C-index were performed based on Kang et al. [35]. *P* values were calculated using the z-score test.

All *P* values were 2-sided with statistical significance set at < 0.05. Data analyses were performed using the DNAnexus platform and RStudio version 4.4.2.

## Results

At baseline, there were 488,223 participants with eBMD assessed. After excluding 276,637 participants aged < 60 years, 160 with dementia at baseline, and 80,396 with missing data on covariates, a total of 131,030 participants were included in the analysis (Supplementary File, Figure S1). Baseline characteristics of the participants are provided in Table 1 (the characteristics of participants grouped by eBMD tertiles are presented in Supplementary File, Table S4). Of the included participants, 68,231 (52.1%) were women, and the median [IQR] age was 64 [62–66] years. There were 4,572 cases (3.5%) of incident dementia and more than 1,916,909 person-years of follow-up (median follow-up time [IQR], 15.4 [14.5–16.2] years).

**Table 1:** Baseline characteristics of participants

	Incident dementia (*n* = 4,572)	No incident dementia (*n* = 126,458)
**Follow-up time**,** median (IQR)**,** y**	10.7 (8.2 to 12.4)	15.4 (14.6 to 16.2)
**Age**,** median (IQR)**,** y**	66 (64 to 68)	64 (62 to 66)
**Sex**,** No. (%)**		
Female	2,143 (46.9)	66,088 (52.3)
Male	2,429 (53.1)	60,370 (47.7)
BMI^*^, **median (IQR)**,** kg/m**^**2**^	26.7 (24.2 to 29.5)	26.7 (24.3 to 29.5)
***APOE*allele status**,** No. (%)**		
High risk	2,584 (56.5%)	34,646 (27.4)
Neural risk	1,696 (37.1)	75,234 (59.5)
Low risk	292 (6.4)	16,578 (13.1)
**Education**^†^, **No. (%)**		
Higher	1,712 (37.4)	55,570 (43.9)
Secondary	1,397 (30.6)	41,740 (33.0)
Vocational/Other	1,463 (32.0)	29,148 (23.0)
**Ethnicity**,** No. (%)**		
White	4,407 (96.4)	122,661 (97.0)
Non-white	165 (3.6)	3,797 (3.0)
**Socioeconomic status quintile** ^‡^		
1 (least deprived)	903 (19.8)	27,049 (21.4)
2–4	2,642 (57.8)	76,961 (60.9)
5 (most deprived)	1,027 (22.5%)	22,448 (17.8)
**Smoking status**,** No. (%)**		
Never	2,115 (46.3)	63,967 (50.6)
Previous	2,102 (46.0)	53,785 (42.5)
Current	355 (7.8)	8,706 (6.9)
**Alcohol intake status**,** No. (%)**		
Never	289 (6.3)	5,222 (4.1)
Previous	245 (5.4)	4,020 (3.2)
Current	4,038 (88.3)	117,216 (92.7)
**Physical activity level**,** No. (%)**		
Low (< 150 min/week)	657 (14.4)	18,503 (14.6)
High (≥ 150 min/week)	3,915 (85.6)	107,955 (85.4)
**Chronic Conditions**,** No. (%)**		
Stroke	205 (4.5)	2,389 (1.9)
Diabetes	524 (11.5)	7,438 (5.9)
Hypertension	1,887 (41.3)	43,305 (34.2)
Arthritis	98 (2.1)	22,491 (17.8)
Rheumatoid arthritis	962 (21.0)	1,801 (1.4)
**Chronic condition present**^§^, **No. (%)**	2,647 (57.9)	60,687 (48.0)
**eBMD**,** median (IQR)**,** g/cm**^**2**^	0.515 (0.433 to 0.607)	0.518 (0.440 to 0.607)

Note: Percentages may not add up to 100 due to rounding

Abbreviations: APOE, apolipoprotein E; eBMD, estimated bone mineral density; IQR, interquartile range

^*^BMI: body mass index, calculated as weight in kilograms divided by height in meters squared

^†^Education: higher education defined as college/university degree or other professional qualification; secondary education defined as first and second/final stage of secondary education; vocational education/other defined work-related practical qualifications and other qualifications

^‡^Socioeconomic status: classified based on Townsend deprivation index, quintiles 1 as least deprived, quintile 2–4 and quintile 5 as most deprived, combining information on social class, employment, car availability, and housing

^§^Chronic condition present indicate that a physician had diagnosed at least one of the chronic conditions, including stroke, diabetes, hypertension, arthritis (identified by ICD-10 M15-M1) and rheumatoid arthritis (identified by ICD-10 M05/M06)

**Fig. 1:**
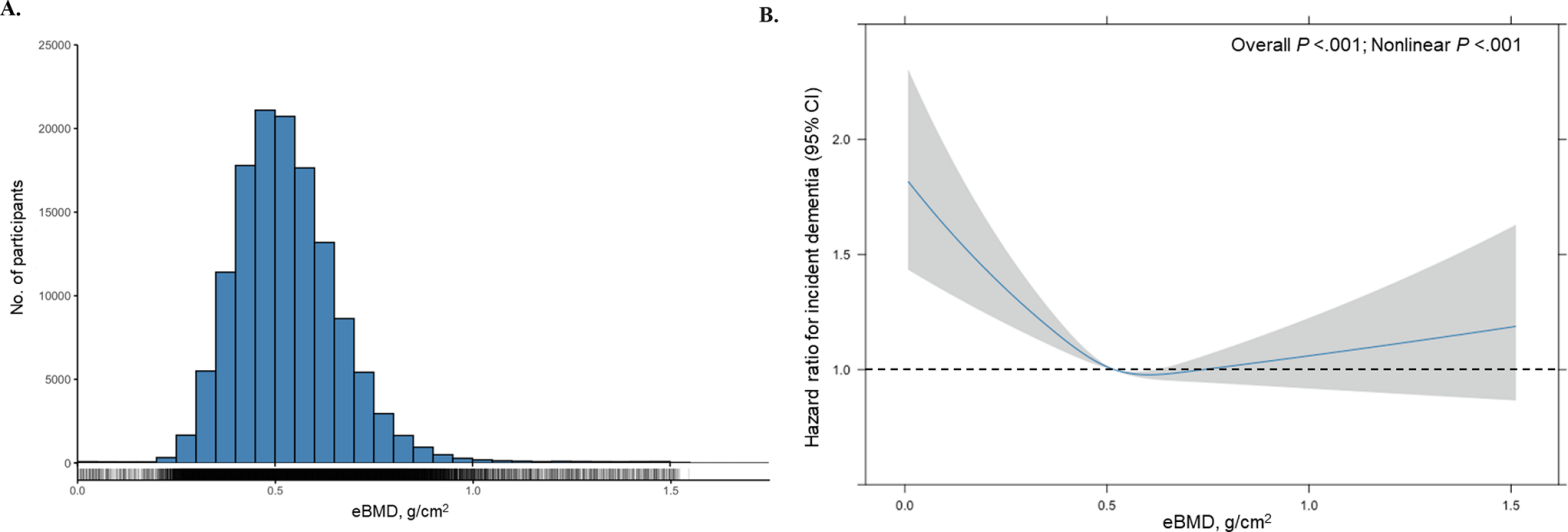
Associations of eBMD and incident dementia. **A**. Distribution of eBMD values and dementia cases. Histogram shows participant counts across the range of eBMD values and vertical lines stand for dementia cases. **B**. Fully adjusted association model. Linear Akaike information criterion is 101485.1 and nonlinear Akaike information criterion is 101473.5 (χ² =17.59, *P* <.001), indicating that nonlinear model best described the relationship between eBMD and incident dementia. The reference value (HR = 1; horizontal dash line) was set at the median of eBMD (0.518 g/cm^2^). The shaded areas reflect the 95% CIs for the HRs. The model was fully adjusted for age, sex, *APOE* genetic risk and additional covariates including body mass index, education level, ethnicity, socioeconomic status, smoking status, alcohol intake status, physical activity level, chronic conditions

**Fig. 2:**
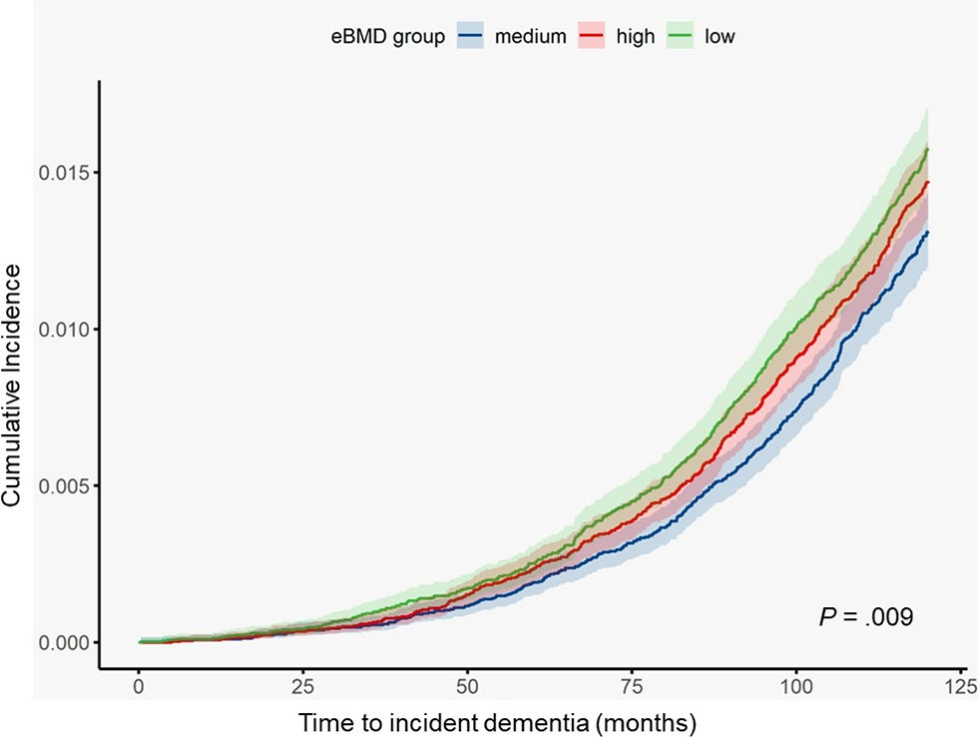
Kaplan-Meier curves for incident dementia diagnosis according to eBMD tertiles. The cumulative incidence of each group was computed by competing risk regression model and was compared using log-rank test, which showed significant difference among the groups (*P* <.05)

The distribution of eBMD was approximately normal, with most participants having eBMD values centred around 0.500 g/cm^2^ (Fig. 1A). The fully adjusted nonlinear model revealed a U-shaped relationship between eBMD with incident dementia (Overall *P* <.001, nonlinear *P* <.001; Fig. 1B). Generally, compared to the medium exposure level, participants with lower or higher exposure level of eBMD had increasing risk of developing incident dementia. This pattern was consistent across both minimally and fully adjusted models, with the tertiles of eBMD used as the exposure. In the linear models using eBMD tertiles, the medium tertile served as the reference group (0.467-<0.573 g/cm^2^ of eBMD). The incidence rate of dementia (cases per 1000 person-years) was 2.51 for the lowest tertile, 2.28 for the medium tertile, and 2.37 for the highest tertile. After minimal adjustment for age and sex, participants in the lowest tertile (< 0.467 g/cm^2^) had a 14% increase in the rate of dementia incidence (HR 1.14, 95% CI 1.06–1.23; *P* <.001), whereas no significant difference was observed for participants with the highest tertile of eBMD (HR 1.00, 95% CI 0.93–1.08; *P* =.93) (Table 2). When eBMD was analysed as a continuous variable, each standard deviation (SD) decrease in eBMD was associated with a 49% increase in dementia risk (HR 1.49, 95% CI 1.19–1.86; *P* <.001). While these effects were slightly attenuated after further adjustment for potential confounders, there remained evidence that lower eBMD was independently associated with increased dementia risk, regardless of genetic predisposition and other covariates (Table 2). Cumulative incidence curves for dementia diagnoses, stratified by eBMD tertiles, initially overlapped. However, over time, the curve for participants in the lowest eBMD tertile rose more rapidly than those in the medium and high tertiles (*P* =.009) (Fig. 2).

**Table 2:** Risk of incident dementia according to eBMD tertiles

	eBMD by tertile							
	Lowest tertile (< 0.467 g/cm^2^)		Medium tertile (0.467-<0.573 g/cm^2^)		Highest tertile (≥ 0.573 g/cm^2^)		Per SD decrease	
Incident dementia								
No. of cases	1,599		1,461			1,512		4,572
Person-years	637,796		641,348			637,765		1,916,909
Cases per 1000 person-years	2.51		2.28			2.37		2.39
Minimally adjusted model^*^								
HR (95% CI)	**1.14 (1.06–1.23)**		1 [Reference]			1.00 (0.93–1.08)		**1.49 (1.19–1.86)**
* P* value	**< 0.001**					0.93		**< 0.001**
Fully adjusted model^†^								
HR (95% CI)	**1.12 (1.05–1.21)**		1 [Reference]			1.01 (0.94–1.09)		**1.39 (1.11–1.73)**
*P* value	**0.001**					0.69		**0.004**

Abbreviations: HR, hazard ratio; eBMD, estimated bone mineral density. SD, standard deviation

Bold font corresponds to significant *P* value threshold

^*^Adjusted for age, sex and *APOE* genetic risk status

^†^Adjusted for age, sex, *APOE* genetic risk and additional covariates including body mass index, education level, ethnicity, socioeconomic status, smoking status, alcohol intake status, physical activity level, chronic conditions

**Fig. 3:**
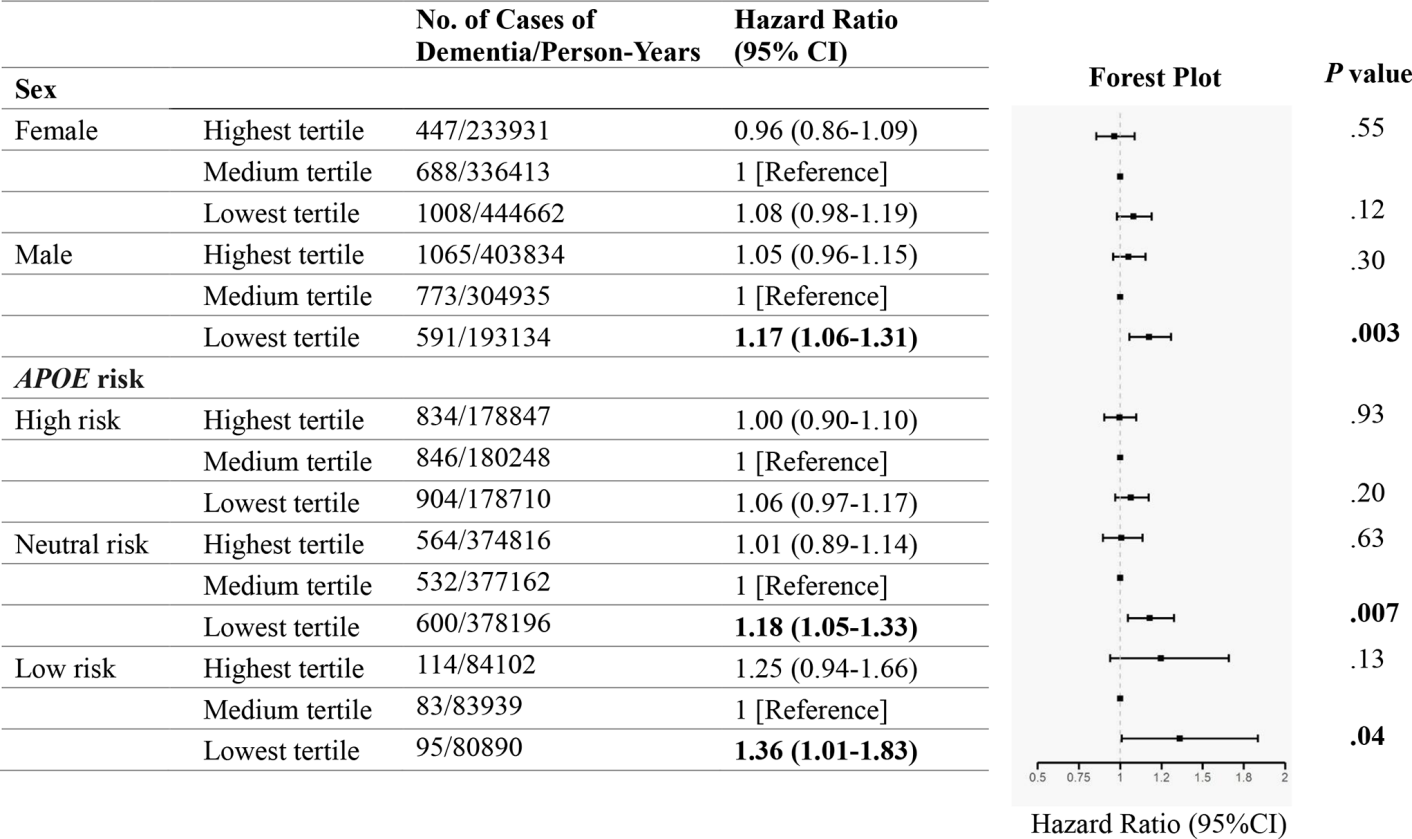
Risk of incident dementia according to sex and *APOE* genetic risk status. Cox proportional-hazards model was used to investigate the association between eBMD and incident dementia stratified by sex and *APOE* genetic risk. The model was fully adjusted for age, sex, *APOE* genetic risk and additional covariates including body mass index, education level, ethnicity, socioeconomic status, smoking status, alcohol intake status, physical activity level, chronic conditions. *P* value for overall interaction between eBMD and sex by Wald test is 0.021, *P* value for overall interaction between eBMD and *APOE* genetic risk by Wald test is 0.14. Bold font corresponds to significant *P* value threshold

There was statistical evidence suggesting an interaction between eBMD with sex (*P* =.021) on the risk of developing dementia. Further stratification analyses of sex showed that eBMD was associated with increased dementia risk only in male participants with lowest tertile of eBMD (HR 1.17, 95% CI 1.06–1.31; *P* =.003), but not in male participants with highest tertile of eBMD or in all female participants (Fig. 3). Notably, high eBMD showed protective value for female elders, although it is non-significant (HR 0.96, 95% CI 0.86–1.09; *P* =.55). When stratified by *APOE* genetic risk, no significant difference on the risk of incident dementia was found between participants with highest and medium tertiles of eBMD across all *APOE* genetic risk categories. For participants with lowest tertile of eBMD, a 18% (HR 1.18, 95% CI 1.05–1.33; *P* =.007) and a 36% (HR 1.36, 95% CI 1.01–1.83; *P* =.04) increase on the risk of incident dementia was found among neutral genetic risk and low genetic risk categories, respectively, when compared to those with medium tertile of eBMD (Fig. 3). There was no statistical evidence of an interaction between eBMD and *APOE* genetic risk (*P* =.14), indicating that the association between eBMD and incident dementia did not vary on the basis of genetic risk.

In the sensitivity analyses, a nonlinear association between eBMD with incident dementia was observed when using imputed data for missing covariates (Supplementary File, Figure S2). A similar pattern of associations between eBMD with incident dementia was found as in the main analyses with the tertiles of eBMD as an exposure with the imputed dataset (Supplementary File, Table S5), and stratification by age groups (Supplementary File, Table S6). Specifically, when stratifying participants by follow-up periods, the effects of lowest tertile of eBMD on dementia incidence were found to be stronger in the first 5-year follow-up (HR 1.41, 95% CI 1.07–1.87; *P* =.016) compared to 5–10 years (HR 1.13, 95% CI 1.00-1.27; *P* = .06) and over 10 years follow-up (HR 1.06, 95% CI 0.99–1.10; *P* =.21) (Supplementary File, Table S7). The same pattern was observed for the highest tertile of eBMD, identifying the early risk for incident dementia within 5 years. Additional adjustment for serum phosphate levels did not materially alter the association between eBMD tertiles and incident dementia (Supplementary File, Table S8), suggesting that the observed relationship is independent of phosphate metabolism.

Finally, we evaluated whether adding eBMD improved the prediction of incident dementia beyond the base models. While the C-index values from the Cox regression models showed statistically significant differences with the inclusion of eBMD (*P* <.001), the overall improvement in predictive performance was minimal over the follow-up period (Supplementary File, Table S9). These findings suggest that while eBMD is associated with dementia risk, its added value for prediction beyond established risk factors is limited.

## Discussion

Our findings show that heel eBMD was independently associated with all-cause incident dementia among older adults, with a nonlinear relationship observed. Lower eBMD levels were linked to an increased risk of future dementia diagnosis, particularly within the first 5 years of follow-up, even after adjusting for major sociodemographic, lifestyle, and genetic factors. Additionally, a similar effect of lower eBMD on incident dementia was observed across all stratified categories by sex and *APOE* status, suggesting that the main effects of eBMD better explain the impact on dementia risk.

Numerous studies have examined the relationship between DXA-BMD and the risk of dementia, demonstrating that lower BMD at the femoral neck is associated with an increased risk of dementia and AD [7, 8, 9, 10, 11, 12]. However, to our knowledge, no prior research has explored the association between heel eBMD and the incidence of all-cause dementia in older populations. A recent study utilizing data from the UK Biobank reported that higher bone density assessed using the QUS method, was associated with a lower rate of neurodegenerative diseases (HR for high exposure level: 0.94, 95% CI 0.89–0.99), compared to lower exposure levels [36]. While this study observed similar protective effects of eBMD, it did not specifically evaluate the relationship between eBMD and incident dementia. Instead, it focused on a disease cluster encompassing both AD and Parkinson’s disease, which have distinct pathogenetic mechanisms. Moreover, that study included participants across all age groups, including individuals under 60 years old, whereas dementia predominantly affects older adults. In contrast, by exclusively including individuals aged 60 years and older and defining dementia as the endpoint, our study provides statistical evidence that lower eBMD correlates with increasing risk of incident dementia. Notably, while high DXA-measured BMD has been identified as a protective factor against dementia, we observed a slight, nonsignificant increase in hazard for high eBMD compared to medium eBMD, particularly in the male population. This discrepancy may be attributable to methodological differences between the DXA and QUS studies.

Moreover, although interactions were observed between eBMD and sex, but not between eBMD and *APOE* status, our findings revealed comparable effects of both sex and *APOE* status on eBMD as previously reported for DXA-BMD [10]. Specifically, lower eBMD was associated with an increased risk of dementia in male but not in female participants. Notably, high *APOE* genetic risk was found to attenuate the protective effects of eBMD on dementia, aligning with findings regarding the impact of *APOE*-ε4 carrier status on DXA-BMD. However, when the *APOE*-ε4 non-carrier group was further subdivided into neutral and low genetic risk categories, lower *APOE* genetic risk was found to amplify the impact of low eBMD on incident dementia risk. These findings contribute new insights to the growing body of evidence that heel eBMD, as measured by QUS, is associated with incident dementia in a pattern consistent with that observed for DXA-BMD. Further research should investigate whether eBMD is associated with structural brain changes, as has been demonstrated for DXA-BMD, such as reductions in white matter volume and increases in white matter hyperintensity volume [37, 38, 39]. Such studies could strengthen the evidence linking eBMD to the cognitive decline observed in dementia progression.

Hip fracture is commoner in patients with dementia, and is associated with an increased risk of future dementia [40, 41]. QUS measurements can predict the risk of hip and non-spine fractures in older men and women [19, 20]. One possible explanation is that low eBMD is a marker of fragility of bone, therefore, individuals who have lower eBMD are more likely to experience hip fracture, and are also at higher risk of developing dementia in later life. Another possible explanation for this is that there are potential factors that can contribute to both bone loss and cognitive decline [42]. We previously reported that blood levels of the bone-derived protein, sclerostin which is mainly produced by osteocytes, were elevated in individuals with positive β-amyloid status on brain PET scan [5]. The plasma level of sclerostin has been found to increase during ageing, and the protein inhibits bone formation by suppressing Wnt/β-catenin signalling, normal activity of which is also vital for maintaining brain function. We therefore speculated that sclerostin might also play a role in the progression of AD, and thus leading to the comorbidity of bone loss and cognitive dysfunction. This speculation was later supported by the study from Shi. Y et al., which demonstrated that sclerostin produced by osteocytes can traverse the blood brain barrier and contribute to the pathological changes of AD [6]. However, the relationship between DXA-BMD and blood sclerostin levels varies by condition [43, 44, 45], and the correlation between sclerostin and eBMD has not been determined. Therefore, further studies would be warranted to investigate whether elevated sclerostin levels correlate with lower eBMD in the UK Biobank cohort.

Our discriminative analyses revealed that adding eBMD to the base models resulted in statistically significant differences in the C-index, however, the minimal increase in predictive accuracy suggests that eBMD alone may not be a strong standalone biomarker for dementia risk stratification in clinical settings. While lower eBMD is associated with dementia risk, its predictive value beyond traditional factors such as age, sex, and *APOE* genetic risk status appears limited. This may be due to the influence of various systemic factors on bone health, including physical activity, hormonal changes, and chronic diseases, which could reduce its specificity for dementia prediction. Despite these limitations, our findings reinforce the growing link between skeletal health and neurodegeneration. Future research should explore whether integrating multiple bone-related biomarkers with genetic and neuroimaging data enhances dementia prediction models. Additionally, longitudinal studies assessing whether interventions targeting bone health (e.g., osteoporosis treatments) influence dementia risk, and could provide further insight into potential causal relationships.

Limitations.

The present study has strengths of large sample size, extended follow-up time over 10 years from middle-life to late-life, and adjustment for potential confounders. Despite these strengths, several limitations need to be acknowledged. First, due to the retrospective and observational nature of this study, we are unable to draw any causal conclusions. The association between eBMD and incident dementia unravelled here cannot support an etiologic link between these two. Second, longitudinal changes in eBMD were not monitored. Although there were two additional follow-ups for eBMD measurement at 2012–2013 and 2014–2016, data from only 19,586 and 6,987 individuals respectively was collected; and fewer than one hundred incident dementia cases completed all three follow-ups, which limits further analyses determining the relationship between trajectory of eBMD changes and incident dementia. Third, only eBMD of the left heel was recorded at baseline for the UK Biobank cohort, and whether eBMD of the right heel has the same pattern of association with incident dementia is unknown. Fourth, information regarding consumption of drugs that may affect bone metabolism [46], such as hormones, steroids, bisphosphonates and calcitonin, was not included in confounders, which could influence the findings. Fifth, our findings are limited to the consenting UK-based cohort which is likely to be healthier and better educated than the general population [47, 48], and the participants were primarily of European origin, which might restrict the generalizability of our findings to other populations. Finally, given that DXA-BMD is the gold standard for diagnosing osteoporosis, a study comparing the association of DXA-BMD and eBMD with incident dementia would be ideal to confirm the relationship between bone health and cognitive function.

## Conclusions

This large cohort study provides robust evidence of an association between lower eBMD in the heel and higher incidence of all-cause dementia in an ageing population. Our findings highlight the potential and suitability of eBMD as a screening technique which can be readily performed in a primary care setting, and could be part of a program for identifying older adults at increased risk of developing dementia. Further research investigating the role of bone health on the progression of dementia will be beneficial for determining whether the association between reduced eBMD and dementia risk has a causal basis, and to aid in the development of effective preventative strategies for delaying or preventing dementia onset.

## Electronic supplementary material

Below is the link to the electronic supplementary material.

Supplementary Material 1

## Data Availability

The datasets analysed during the current study are available in the UB Biobank through application, https://www.ukbiobank.ac.uk/.
