# Supplementary material for: Association of heel bone mineral density with incident dementia among ageing adults: a population-based study from the UK Biobank
Source: Aging Clin Exp Res. 2025 Jul 16;37(1):217. doi: 10.1007/s40520-025-03100-w (PMC12267336; doi:10.1007/s40520-025-03100-w)
Supplement: Supplementary file 1 — Supplementary Material 1 [file 40520_2025_3100_MOESM1_ESM.docx]

**Supplementary File**

**Figure S1** Flowchart of study participant selection process.

**Figure S2** Nonlinear association between risk of incident dementia and eBMD after imputation for missing data.

**Table S1** Code used by UK Biobank for diagnosing all-cause dementia.

**Table S2** *APOE* SNP alleles and frequency of *APOE* genotypes (N=131,030).

**Table S3** Proportion of missing values for each variable.

**Table S4** Cohort characteristics by eBMD tertiles.

**Table S5** Risk of incident dementia according to eBMD tertile for imputed dataset.

**Table S6** Cox proportional-hazards models investigating the association between eBMD and incident dementia among different age groups (60-65 and over 65).

**Table S7** Cox proportional-hazards models investigating the association between eBMD and incident dementia among follow-up time groups (0-5y, 5-10y and over 10-y).

**Table S8** Risk of incident dementia according to eBMD tertiles with serum phosphate level as additional covariate (N = 114357).

**Table S9** C-index from Cox regression models using eBMD for incident dementia discrimination.

**Figure S1** Flowchart of study participant selection process.

Participants in the UK Biobank with baseline eBMD data (N= 488,223)

Excluded:

- Participants aged <60 years (n=276,637)
- Participants with prevalent dementia (n=160)

Participants with complete covariate data

n=131,030 (61.97%)

Participants with ≥1 missing covariate data

n=80,396 (38.03%)

Multiple Imputation Analysis

n=211,426

Final analyses

n=131,030

**Figure S2** Nonlinear association between risk of incident dementia and eBMD after imputation for missing data.


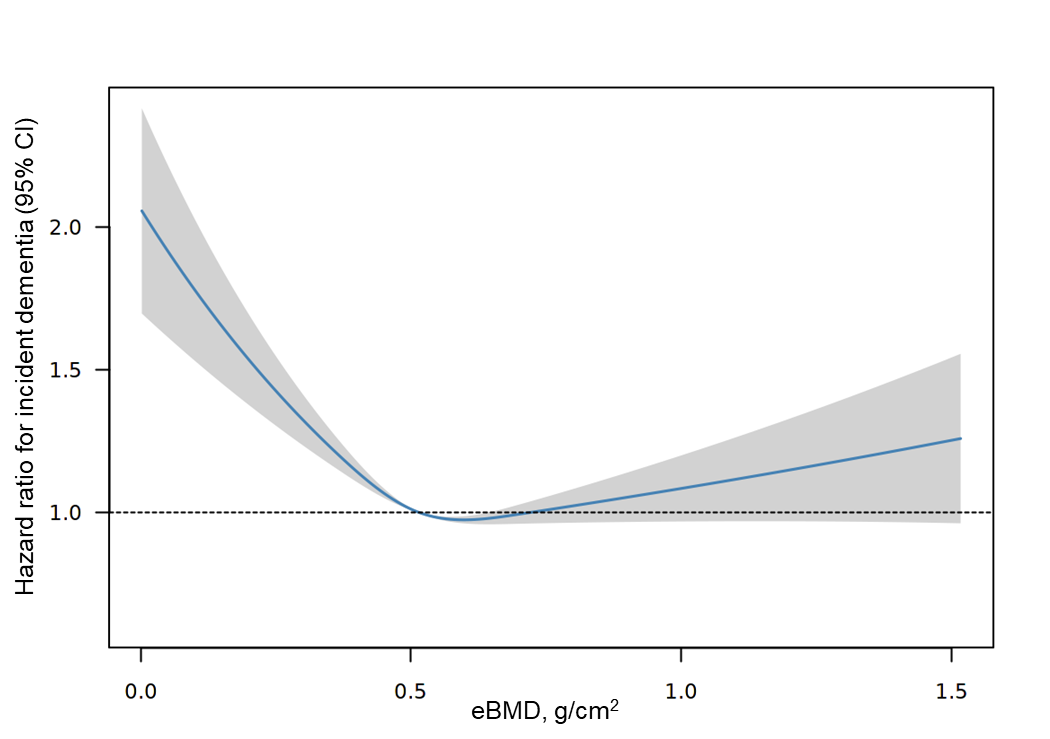


HRs were the average value for 40 imputed datasets. The reference value (HR = 1; horizontal dash line) was set at the median of eBMD (0.518 g/cm^2^). There were Shaded area represents 95% confidence intervals. There were 203,268 non-incident dementia and 8,158 dementia cases in each imputed dataset. Cox model was fully adjusted for age, sex, *APOE* genetic risk and additional covariates including body mass index, education level, ethnicity, socioeconomic status, smoking status, alcohol intake status, physical activity level, chronic conditions.

**Table S1** Code used by UK Biobank for diagnosing all-cause dementia.

| **Disease** | **ICD-9 codes** | **ICD-10 codes** | **Self-Reported codes** |
| --- | --- | --- | --- |
| AD | 331.0 | F00, F00.0, F00.1, F00.2, F00.9, G30, G30.0, G30.1, G30.8, G30.9 | Field 20002  Code 1263 |
| VD | 290.4 | F01, F01.0, F01.1, F01.2, F01.3, F01.8, F01.9, I67.3 |  |
| FTD | 331.1 | F02.0, G31.0 |  |
| Other types of dementia | 290.2, 290.3, 291.2, 294.1, 331.2, 331.5 | A81.0, F02, F02.1, F02.2, F02.3, F02.4, F02.8, F03, F05.1, F10.6, G31.1, G31.8 |  |

Abbreviations: ICD, International Classification of Diseases; AD, Alzheimer disease; VD, vascular dementia; FTD, frontotemporal dementia.

**Table S2** *APOE* SNP alleles and frequency of *APOE* genotypes (N=131,030).

| ***APOE* variant** | **SNP alleles** | |
| --- | --- | --- |
|  | ***rs429358*** | ***rs7412*** |
| ε1 | C | T |
| ε2 | T | T |
| ε3 | T | C |
| ε4 | C | C |

| ***APOE risk*** | ***APOE* Genotype** | ***rs429358*** | ***rs7412*** | **Count, n. (%)** |
| --- | --- | --- | --- | --- |
| High | ε4ε4 | CC | CC | 3138 (2.39%) |
|  | ε3ε4 | TC | CC | 30806 (23.51%) |
|  | ε2ε4 | TC | TC | 3286^*^ (2.51%) |
|  | ε1ε3 | CT | TC |  |
| Natural | ε3ε3 | TT | CC | 76930 (58.71%) |
| Low | ε2ε3 | TT | TC | 16115 (12.30%) |
|  | ε2ε2 | TT | TT | 707 (0.54%) |
|  | ε2ε2/ε1ε1/ε1ε2^†^ | untyped | TT | 48 (0.04%) |
| Unsure^‡^ | Cases with genotypes of uncertain risk (ε1ε4) or without SNP data | | | |

^*^Ambiguous genotypes, ε2ε4 and ε1ε3, were coded as ε2ε4 since the ε1 allele is too rare.

^†^rs429358 is untyped and rs7412=TT, these cases could be ε2ε2, ε1ε1 or ε1ε2 and were coded as ε2ε2 since the ε1 allele is too rare.

^‡^Participants with uncertain *APOE* status were assigned to “Unsure” and were excluded from main analyses.

**Table S3** Proportion of missing values for each variable.

| **Variables** | **Missing rate (%)** |
| --- | --- |
| *APOE* genetic risk | 17.43 |
| BMI | 0.19 |
| Ethnicity | 0.37 |
| Education | 1.36 |
| Socioeconomic status | 0.08 |
| Smoking status | 0.60 |
| Alcohol intake | 0.21 |
| Physical activity | 22.12 |
| Chronic conditions | 0.89 |

Overall proportion for missing values was 38.03%, and therefor 40 imputations were used for imputation analyses.

**Table S4** Cohort characteristics by eBMD tertiles.

|  | **Lowest tertile** | **Medium tertile** | **Highest tertile** |
| --- | --- | --- | --- |
| **Dementia cases/Total** | 1,599/43,742 | 1,461/43,765 | 1,512/43,523 |
| **Age, median (IQR), y** | 64 (62 to 67) | 64 (62 to 66) | 64 (62 to 66) |
| **Sex, No. (%)** | | |  |
| Female | 30,022 (68.6) | 22,553 (51.5) | 15,656 (36.0) |
| Male | 13,720 (31.4) | 21,212 (48.5) | 27,867 (64.0) |
| **BMI^*^, median (IQR), kg/m^2^** | 26.02  (23.61 to 28.91) | 26.89  (24.54 to 29.71) | 27.12  (24.81 to 29.94) |
| ***APOE* allele status, No. (%)** | | |  |
| High risk | 12,423 (28.4) | 12,448 (28.4) | 12,359 (28.4) |
| Neural risk | 25,821 (59.0) | 25,640 (58.6) | 25,469 (58.5) |
| Low risk | 5,498 (12.6) | 5,677 (13.0) | 5,695 (13.1) |
| **Education^†^, No. (%)** | | |  |
| Higher | 18,010 (41.2) | 19,142 (43.7) | 20,130 (46.3) |
| Secondary | 14,994 (34.3) | 14,494 (33.1) | 9,744 (22.4) |
| Vocational/Other | 10,738 (24.5) | 10,129 (23.1) | 13,649 (31.4) |
| **Ethnicity, No. (%)** | | |  |
| White | 42,791 (97.8) | 42,485 (97.1) | 41,792 (96.0) |
| Non-white | 951 (2.2) | 1,280 (2.9) | 1,731 (4.0) |
| **Socioeconomic status quintile**^‡^ | | |  |
| 1 (least deprived) | 8,972 (20.5) | 9,283 (21.2) | 9,697 (22.3) |
| 2 - 4 | 26,384 (60.3) | 26,683(61.0) | 26,536 (61.0) |
| 5 (most deprived) | 8,386 (19.2) | 7,799 (17.8) | 7,290 (16.7) |
| **Smoking status, No. (%)** | | |  |
| Never | 22,463 (51.4) | 21,817 (49.9) | 21,802 (50.1) |
| Previous | 17,870 (40.9) | 18,991 (43.4) | 19,026 (43.7) |
| Current | 3,409 (7.8) | 2,957 (6.8) | 2,695 (6.2) |
| **Alcohol intake status, No. (%)** |  |  |  |
| Never | 2,116 (4.8) | 1,847 (4.2) | 1,548 (3.6) |
| Previous | 1,596 (3.6) | 1,408 (3.2) | 1,261 (2.9) |
| Current | 40,030 (91.5) | 40,510 (92.6) | 40,714 (93.5) |
| **Physical activity level, No. (%)** | | |  |
| Low (<150 min/week) | 6,770(15.5) | 6,558 (15.0) | 5,832 (13.4) |
| High (≥150 min/week) | 36,972 (84.5) | 37,207 (85.0) | 37,691 (86.6) |
| **Chronic Conditions, No. (%)** | | |  |
| Stroke | 885 (2.0) | 827 (1.9) | 882 (2.0) |
| Diabetes | 1,994 (4.6) | 2,728 (6.2) | 3,240 (7.4) |
| Hypertension | 13,930 (31.8) | 15,406 (35.2) | 15,856 (36.4) |
| Arthritis | 7,990 (18.3) | 7,711 (17.6) | 7,752 (17.8) |
| Rheumatoid arthritis | 858 (2.0) | 582 (1.3) | 459 (1.1) |
| **Chronic Condition Present**^§^, **No. (%)** | 20,213 (46.2) | 21,291 (48.6) | 21,830 (50.2) |
| **eBMD, median (IQR), g/cm^2^** | 0.409 (0.368 to 0.440) | 0.518 (0.493 to 0.545) | 0.649 (0.607 to 0.716) |

Note: Percentages may not add up to 100 due to rounding.

Abbreviations: APOE, apolipoprotein E; eBMD, estimated bone mineral density; IQR, interquartile range.

^*^BMI: body mass index, calculated as weight in kilograms divided by height in meters squared.

^†^Education: higher education defined as college/university degree or other professional qualification; secondary education defined as first and second/final stage of secondary education; vocational education/other defined work-related practical qualifications and other qualifications.

^‡^Socioeconomic status: classified based on Townsend deprivation index, quintiles 1 as least deprived, quintile 2–4 and quintile 5 as most deprived, combining information on social class, employment, car availability, and housing.

^§^Chronic condition present indicate that a physician had diagnosed at least one of the chronic conditions, including stroke, diabetes, hypertension, arthritis (identified by ICD-10 M15-M1) and rheumatoid arthritis (identified by ICD-10 M05/M06).

**Table S5** Risk of incident dementia according to eBMD tertiles for imputed dataset.

|  | eBMD by tertile | | |  | |
| --- | --- | --- | --- | --- | --- |
|  | **Lowest tertile**  **(<0.464 g/cm^2^)** | **Medium tertile**  **(0.464-<0.570 g/cm^2^)** | **Highest tertile**  **(≥0.570 g/cm^2^)** | | **Per SD decrease** |
| Incident dementia | | | | |  |
| No. of cases | 2913 | 2554 | 2691 | | 8158 |
| Person-years | 1016886 | 1023889 | 1023588 | | 3064363 |
| Cases per 1000 person-years | 2.86 | 2.49 | 2.63 | | 2.67 |
| Minimally adjusted model^*^ |  |  |  | |  |
| HR (95% CI) | **1.18 (1.12-1.25)** | 1 [Reference] | 1.02 (0.96-1.07) | | **1.57 (1.33-1.85)** |
| *P* value | **<.001** |  | .59 | | **<.001** |
| Fully adjusted model^†^ |  |  |  | |  |
| HR (95% CI) | **1.17 (1.11-1.23)** | 1 [Reference] | 1.02 (0.97-1.08) | | **1.47 (1.25-1.73)** |
| *P* value | **<.001** |  | .42 | | **<.001** |

Abbreviations: HR, hazard ratio; eBMD, estimated bone mineral density. SD, standard deviation.

Bold font corresponds to significant *P* value threshold.

^*^Adjusted for age, sex and *APOE* genetic risk status.

^†^Adjusted for age, sex, *APOE* genetic risk and additional covariates including body mass index, education level, ethnicity, socioeconomic status, smoking status, alcohol intake status, physical activity level, chronic conditions.

**Table S6** Cox proportional hazards models investigating the association between eBMD and incident dementia among different age groups (60-65 and over 65).

|  | No. of Cases of  Dementia/Person-Years | Cases per 1000 person-years | Hazard Ratio  (95% CI) | Fully adjusted  *P* Value |
| --- | --- | --- | --- | --- |
| 60-65 years old |  |  |  |  |
| Lowest tertile | 699/423664 | 1.65 | **1.11** (1.00-1.24) | **.048** |
| Medium tertile | 668/436443 | 1.53 | 1 [Reference] |  |
| Highest tertile | 656/433860 | 1.51 | 0.96 (0.86-1.06) | .41 |
| over 65 years old |  |  |  |  |
| Lowest tertile | 900/214132 | 4.20 | **1.12** (1.12-1.23) | **.02** |
| Medium tertile | 793/204905 | 3.87 | 1 [Reference] |  |
| Highest tertile | 865/203905 | 4.20 | 1.07 (0.97-1.18) | .18 |

Bold font corresponds to significant *P* value threshold.

Cox model was fully adjusted for age, sex, *APOE* genetic risk and additional covariates including body mass index, education level, ethnicity, socioeconomic status, smoking status, alcohol intake status, physical activity level, chronic conditions.

**Table S7** Cox proportional hazards models investigating the association between eBMD and incident dementia among follow-up time groups (0-5y, 5-10y and over 10-y).

|  | No. of Cases of  Dementia/Person-Years | Cases per 1000 person-years | Hazard Ratio  (95% CI) | Fully adjusted  *P* Value |
| --- | --- | --- | --- | --- |
| 0-5 years follow-up |  |  |  |  |
| Lowest tertile | 121/216292 | **0.56** | **1.41** (1.07-1.87) | **.016** |
| Medium tertile | 87/216619 | 0.40 | 1 [Reference] |  |
| Highest tertile | 111/215415 | 0.52 | 1.22 (0.92-1.62) | .16 |
| >5 to 10 years follow-up |  |  |  |  |
| Lowest tertile | 1478/419869 | 3.52 | 1.13 (1.00-1.27) | .06 |
| Medium tertile | 1374/421555 | 3.26 | 1 [Reference] |  |
| Highest tertile | 1401/419029 | 3.34 | 1.02 (0.90-1.15) | .79 |
| Over 10 years follow-up |  |  |  |  |
| Lowest tertile | 916/615305 | 1.49 | 1.06 (0.99-1.10) | .21 |
| Medium tertile | 874/620464 | 1.41 | 1 [Reference] |  |
| Highest tertile | 884/617532 | 1.43 | 1.00 (0.91-1.10) | .96 |

Bold font corresponds to significant *P* value threshold.

Cox model was fully adjusted for age, sex, *APOE* genetic risk and additional covariates including body mass index, education level, ethnicity, socioeconomic status, smoking status, alcohol intake status, physical activity level, chronic conditions.

**Table S8** Risk of incident dementia according to eBMD tertiles with serum phosphate level as additional covariate (N = 114,357^‡^).

|  | eBMD by tertile | | | | | | |  |
| --- | --- | --- | --- | --- | --- | --- | --- | --- |
|  | **Lowest tertile**  **(<0.468 g/cm^2^)** | | **Medium tertile**  **(0.468-<0.573 g/cm^2^)** | | **Highest tertile**  **(≥0.573 g/cm^2^)** | | **Per SD decrease** | |
| Incident dementia | | | | | | | |  |
| No. of cases | 1,393 | | 1,275 | | | 1,330 | | 3,998 |
| Person-years | 554,858 | | 641,348 | | | 557,578 | | 1,753,784 |
| Cases per 1000 person-years | 2.51 | | 2.27 | | | 2.39 | | 2.27 |
| Minimally adjusted model^*^ |  |  | |  | | | |  |
| HR (95% CI) | **1.14 (1.06-1.23)** | | 1 [Reference] | | | 1.02 (0.94-1.10) | | **1.44 (1.13-1.82)** |
| *P* value | **<.001** | |  | | | .67 | | **.002** |
| Fully adjusted model^†^ |  |  | |  | | | |  |
| HR (95% CI) | **1.12 (1.04-1.21)** | | 1 [Reference] | | | 1.03 (0.95-1.11) | | **1.34 (1.05-1.69)** |
| *P* value | **.003** | |  | | | .47 | | **.016** |

Abbreviations: HR, hazard ratio; eBMD, estimated bone mineral density. SD, standard deviation.

Bold font corresponds to significant *P* value threshold.

^‡^16,673 individuals were excluded due to missing serum phosphate data.

^*^Adjusted for age, sex and *APOE* genetic risk status.

^†^Adjusted for age, sex, *APOE* genetic risk and additional covariates including body mass index, education level, ethnicity, socioeconomic status, smoking status, alcohol intake status, physical activity level, chronic conditions and serum phosphate level.

**Table S9** C-index from Cox regression models using eBMD for incident dementia discrimination.

| Base model^*^ | Predictor | C-index | | *P* Value^†^ |
| --- | --- | --- | --- | --- |
|  |  | **Base** | **Base + Predictor** |  |
| Age + Sex | eBMD | 0.592 (0.583-0.600) | 0.593 (0.584-0.602) | <.001 |
| Age + Sex + *APOE* | eBMD | 0.694 (0.685-0.703) | 0.695 (0.686-0.703) | <.001 |

Results for max available follow-up time are given in this table.

^*^Base models were adjusted for covariates including body mass index, education level, ethnicity, socioeconomic status, smoking status, alcohol intake status, physical activity level, chronic conditions.

^†^*P* values were calculated using the z-score test.
